# An insight into the bioelectrochemical photoreduction of CO_2_ to value-added chemicals

**DOI:** 10.1016/j.isci.2021.102294

**Published:** 2021-03-09

**Authors:** Priyanka Gupta, Mohammad Tabish Noori, Abraham Esteve Núñez, Nishith Verma

**Affiliations:** 1 Department of Chemical Engineering, Indian Institute of Technology Kanpur, Kanpur, Uttar Pradesh, 208016, India; 2 Bioelectrogenesis (Bioe) research group, Department of Analytical Chemistry, Physical Chemistry and Chemical Engineering, University of Alcala (UAH), 28805 Alcalá de Henares, Madrid, Spain; 3 IMDEA Water, 28805 Alcalá de Henares, Madrid, Spain

**Keywords:** Biochemistry, Microbial Biotechnology, Materials Synthesis, Biomaterials

## Abstract

Goal of sustainable carbon neutral economy can be achieved by designing an efficient CO_2_ reduction system to generate biofuels, in particular, by mimicking the mechanism of natural photosynthesis using semiconducting nanomaterials interfaced with electroactive bacteria (EAB) in a photosynthetic microbial electrosynthesis (PMES) system. This review paper presents an overview of the recent advancements in the biohybrid photoanode and photocathode materials. We discuss the reaction mechanism observed at photoanode and photocathode to enhance our understanding on the solar driven MES. We extend the discussion by showcasing the potential activity of EABs toward high selectivity and production rates for desirable products by manipulating their genomic sequence. Additionally, the critical challenges associated in scaling up the PMES system including the strategies for diminution of reactive oxygen species, low solubility of CO_2_ in the typical electrolytes, low selectivity of product species are presented along with the suggestions of alternative strategies to achieve economically viable generation of (bio)commodities.

## Graphical abstract

**Figure:**
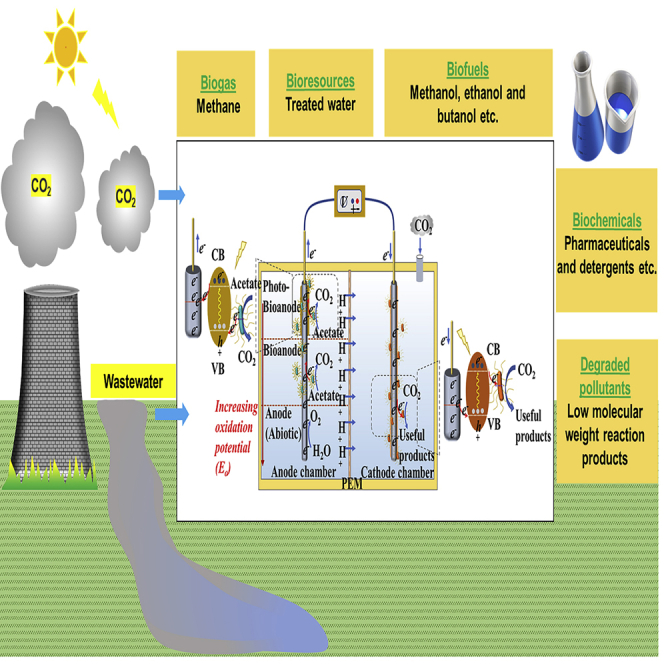

## Introduction

Rapidly increasing concentration of CO_2_ in the atmosphere is a pressing global challenge, which has a direct implication on climate change (Ritchie and Roser, 2017). The use of fossil fuels, as a direct or indirect source of energy and in industrial processes, holds accountable for 65% of the total CO_2_ emission. Therefore, it is highly necessary to develop a deployable carbon neutral technology, capable of recycling CO_2_ to valuable fuels and chemicals in order to prevent environmental deterioration and increase reliance on renewable fuels.

CO_2_ is a stable molecule, and thus, the hydrogenation and chain elongation reaction routes to convert it to biochemicals are invariably energy-intensive processes (Chang et al., 2016; Kumar et al., 2012). For example, conversation of methanol from CO_2_ occurs at 200 – 300°C and 50 – 100 bar pressure on the Cu/ZnO-based catalysts (Nieminen et al., 2019). The energy demand for this reaction is estimated to be 49.8–90 kJ/mol. The light-induced and electrochemical CO_2_ reduction route have also been demonstrated recently (Li and Zhu, 2020; Khan and Tahir, 2019; Goli et al., 2016; Chiranjeevi et al., 2019; Bian et al., 2018). Unfortunately, these methods are not suitable to be considered as a practical technology that can capture and utilize CO_2_. By contrast, researchers have discovered the microbial electrosynthesis (MES) technology—a potential deployable technology—that can recycle CO_2_ to desired biochemical and fuels at room temperature and pressure using electroactive bacteria (EAB) as biocatalyst (Nevin et al., 2010). An MES consists of an anode and a cathode situated in separate anodic and cathodic chamber, respectively, or in a single chamber sharing the electrolyte. In case of a dual chamber MES, a cation exchange membrane (CEM) is generally used to conduct protons from anode to cathode. At the anode, electrons, and protons (H^+^) are generated, which are derived to the cathode via an external imposed electrical potential (Noori and Min, 2019). The electrons transferred to the EABs (e.g., *Moorella thermoacetica, Sporomusa ovata*) assist in converting CO_2_ to the biochemicals via the Wood-Ljungdahl pathway (WLP) (Ragsdale and Pierce, 2008). The WLP uses mainly two enzymes: CO dehydrogenase and acetyl-CoA synthase; the former enzyme helps in reducing CO_2_ and the latter enzyme catalyzes formation of the reactive acetyl-CoA, which acts as a building block for the formation of useful products. The poised cathode potential (vs. standard hydrogen electrode, SHE) is an important factor in MES, and at higher potentials than the cathodic potential a range of product becomes theoretically feasible (Liu et al., 2014; Rabaey and Rozendal, 2010). For example, the MES poised with a cathodic potential of −0.8 V (vs. SHE) could produce a mixture of C4 and C6 carboxylic acids (isobutyric, n-butyric, and n-caproic acids) and the corresponding alcohols (isobutanol, n-butanol, and n-hexanol). However, selectivity of MES depends on the metabolic pathways expressed by the microorganism acting as the biocatalyst (see section 4 for details). Additionally, the required amounts of electric potential can be easily drawn from renewable energy sources, e.g., wind, solar, geothermal, etc. Thus, MES can act as an energy storage device to accumulate the electrical energy produced from various renewable energy sources to chemical energy.

In the very first demonstration of MES, a graphite cathode was interfaced with *S. ovata* biofilm to reduce CO_2_ to acetic acid (Nevin et al., 2010). This research was a model shift towards bioelectrochemical technologies to reduce CO_2_ to multi-carbon organic molecules sustainably. Till then, several approaches were documented to improve the performance and selectivity of the products by developing cutting edge electrode materials, genetically modifying EABs (more details on section 4) and optimizing the dimensions of the system (Shin et al., 2017; Ueki et al., 2014; Banerjee et al., 2014; Noori et al., 2020b; Gil-Carrera et al., 2011). A recent review article covers the various aspects of design and optimization of conventional MESs, which is worth reading (Tufa et al., 2020). In a decade of research and innovation, the researchers have got some earlier success in scaling up MES to a 15-L technology readiness level 4 (TRL 4) reactor, commissioned in IRSTEA, France by Tian et al. (Tian et al., 2019).

**Figure 1:**
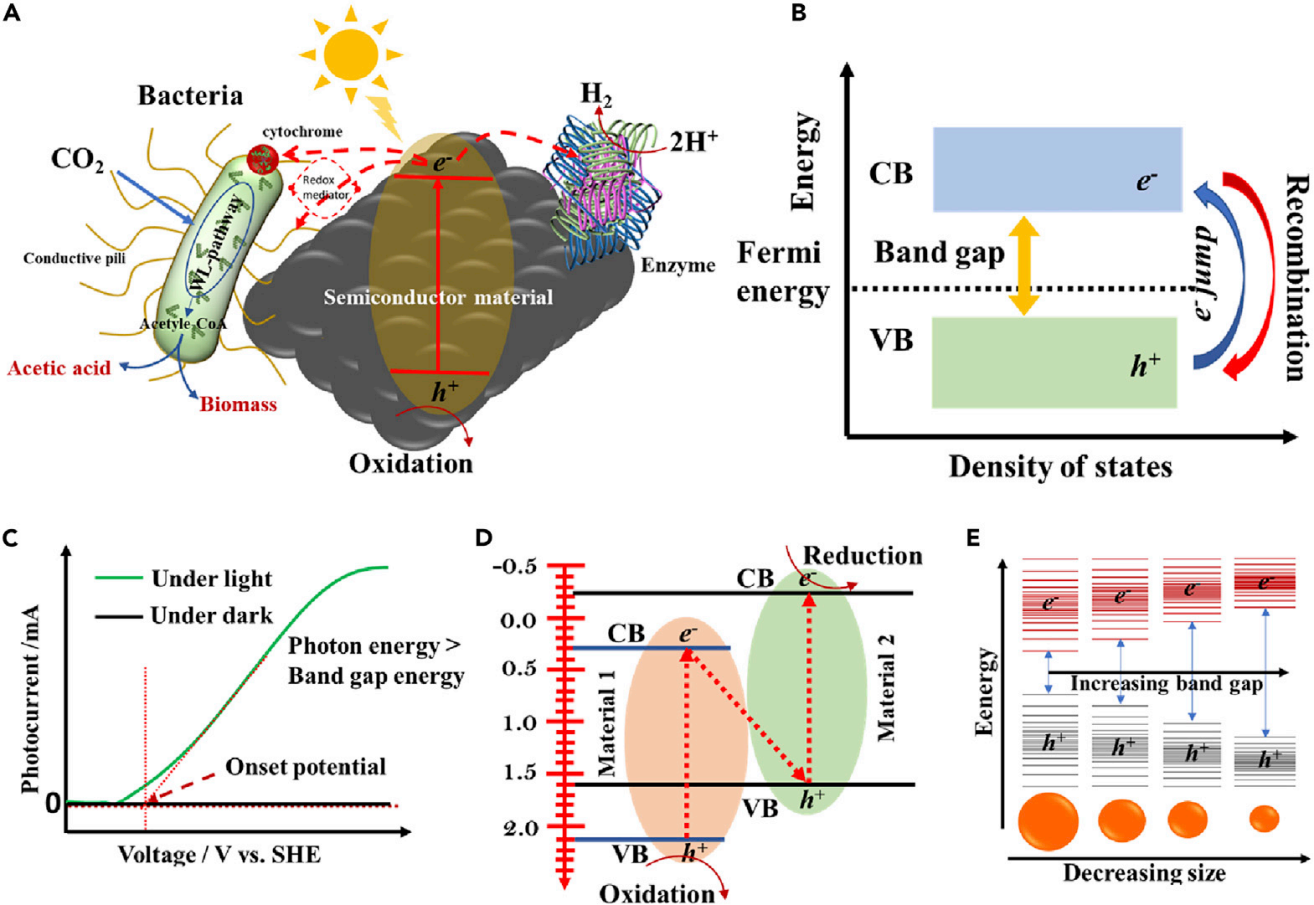
Pictorial representation of different terms frequently used in artificial photosynthesis (A–E) (A) Biohybrid semiconductor materials (left side: EAB with electron transfer and metabolic pathways, and right side: enzymes with electron transfer), (B) important terms in semiconductor materials, such as band gap, Fermi level, electron excitation and recombination, (C) photocurrent vs. voltage graph depicting the photocurrent onset potential measurement technique, (D) Quantum confinement in semiconductor materials, and (E) direct Z-scheme in artificial photosynthesis process.

Biosynthesis of chemicals from CO_2_ as sole feedstock at cathode mainly depends on the extracellular electron transport process occurring between the biocathode and cell-walls of the EABs (Vu et al., 2020a, 2020b). By receiving electrons, EABs convert CO_2_ to reactive acetyl-CoA, followed by oxidation to acetate during ATP phosphorylation (Ragsdale and Pierce, 2008). Hence, limited interaction between the EABs and plain electrode is a critical bottleneck of the process, which significantly decreases the CO_2_-reducing current density. Besides, the loss of electrons during mobility from anode to cathode and cathode to microbe is also a major concern, primarily in ordinary electrode materials. Lately, the nanostructured inorganic semiconductor materials as an electrode have demonstrated significant potential to overcome the above-mentioned drawbacks of the biosynthesis process (Fang et al., 2020c). The application of hybrid semiconductor nanomaterial as the cathode is fascinating because of its enhanced optical and electronic properties. The production of electrons at bioanode via solar energy has also been demonstrated as a preferential technique over abiotic water splitting, which can generate current density at low overpotential (Bian et al., 2020a). Therefore, the interfacing EABs with hybrid semiconductor nanomaterials having low band energy in MES is an artificial photosynthesis technology with high selectivity for CO_2_. By definition, artificial photosynthesis is a chemical process which mimics the natural photosynthesis process to derive valuable chemicals from sunlight, water, and CO_2_ (Figure 1A). Remarkably, solar-driven biosynthesis has shown an astonishing efficiency of ∼ 20%. However, the process efficiency in this case also depends on several factors associated with the electrode materials, e.g., biocompatibility, energy band gap, bandgap alighnement, conductivity, and surface area (Sharma et al., 2019; Lee et al., 2019; Aryal et al., 2017). The bandgap is the energy difference between the valence and conduction bands of a material and band alignment can be defined as the relative alignment of band energy positions close to the interface when two different materials interact with each other (Figure 1B). Recently, different types of hybrid semiconducting nanomaterials have been developed with low band energy, low photocurrent onset potential and high biocompatibility to improve the solar-driven biosynthesis efficacy under visible light irradiation. The photocurrent onset potential is the threshold potential at which photocurrent starts rising from zero value because of the movement of electrons from the valence to conduction band during illumination. It can be calculated by first drawing the maximum slopes at the photocurrent and dark current curves (current vs. voltage), and then finding the intersection of the slopes at x-axis (voltage) (Figure 1C). Most importantly, the materials with ultra-high porosity have shown some interesting features, as each cavity in the electrode framework can act as a nanoreactor to efficiently supply CO_2_ and nutrient to the EABs, which eventually can enhance the MES performance by several folds (Petrosko et al., 2016). However, high efficiency of the system can only be expected at a high cost associated with high purity of the semiconductor nanomaterials. Though, recent research in photo-assisted biosynthesis has demonstrated some promising results, the development of the state-of-the-art solar nanomaterials is still under progress. A clear-cut mechanism of photo-assisted biotransformation of CO_2_ to different biochemicals is yet to be established. The motive of this review paper is to explore two important aspects of the whole-cell EAB-based semi-artificial photosynthesis system, i.e., photosynthetic microbial electrosynthesis (PMES). First, we have explored the most valid and adapted mechanism of the solar assisted biotransformation process occurring at anode and cathode in PMES. Next, recent development of semiconductor nanomaterials-based anode and cathode and their particular features to interact with EABs are discussed in detail. Finally, recent advances in the genetic modulation of EABs to adapt to specific reduction environment as well as to improve the selectivity of the desired products are discussed. The review also contains a way forward section summarizing all the critical challenges related to the development of PMES, and possible future research direction from our point of view. This review article will be helpful to the researchers in understanding the PMES process and critical challenges, and thus, it will be a milestone for future research in this area.

## PMES: mimicking the natural photosynthesis blueprint

Nature has awarded us the plethora of energy, which is around ∼ 1370 W/m^2^ (Johnson, 1954), and therefore, it must be harvested to fulfill the ever-growing global energy demand (Lewis and Nocera, 2006). Natural photosynthesis process is the first ever known blueprint of recycling CO_2_ to biomass, and also, the biggest contributor of CO_2_ fixation. However, this process converts only 1 – 2% of solar energy to biomass. By contrast, the artificial solar harvester (photovoltaic solar cells) composed with the cutting-edge inorganic semiconductor materials are specifically designed to improve the solar harvesting efficiency. As a result, a modern solar panel can achieve an efficiency of 20% (solar to electricity), securing high profitability and a long lasting service (Sahoo et al., 2020). Although having high profit margins, this technology is apparently not fit for CO_2_ mitigation and fixation. Most of the inorganic semiconductor catalysts are either not suitable for CO_2_ reduction or often capable of transforming CO_2_ to C1 compounds such as methanol, formic acid, methane, and carbon monoxide (Barton et al., 2008; Kunene et al., 2019; Schreier et al., 2017). In addition, the conversion efficiency with the state-of-the-art semiconductor nanomaterial is low. This challenge is recently addressed by mimicking the Z-scheme mechanism (Figure 1D) of the natural photosynthesis processes by channeling electrons to the EABs (Liu et al., 2015). The EABs contain multiple metabolic pathways associated with a high number of enzymes and proteins that can easily transform CO_2_ to more reliable low carbon fuels and biochemicals with high selectivity (Cestellos-Blanco et al., 2020).

In biological systems, the intermediately formed activated carbons such as acetyl-CoA and pyruvic acids serve as the reactive building blocks for facilitating the C–C chain elongation, and thus, formation of complex organic molecules occurs (Figure 1). Pure sacrificial enzymes used as a charge carrier for CO_2_ fixation leverage the product selectivity and reduce activation overpotential. For example, Srikanth et al., employed a formate dehydrogenase (FDH) and β-nicotinamide adenine dinucleotide hydrate (NADH) reducing environment in MES to produce formic acid from CO_2_ (Srikanth et al., 2017). The production rate of formic acid increased with addition of FDH and decreased with time because of the lack of recyclability of NADH. When the enzymes were interfaced with a light absorbing cathode, nearly 100% Faradaic efficiency (FE) was achieved. Moreover, the enzymes already have the predefined hydrophilic/hydrophobic sites, and thus, a matching electrode for better binding to the active sites could be easily fabricated.

The application of semiconductor nanomaterials to efficiently capture sunlight and convert it to the usable solar fuel is the recent development in this field. The semiconductor nanomaterials are highly tunable according to their specific application environment having adequate light capturing efficiency, and therefore, they can be easily paired with EABs. Often, these materials support facile charge transfer through conductive layer that can minimize energy loss during microbial metabolism. The very initial model of solar-to-chemical production was an enzymatic hydrogen evolution reaction (HER), which was achieved by suspending hydrogenase (H_2_ase) enzyme in the colloidal solution of the semiconductor nanomaterials such as cadmium sulfide (CdS), CdTe, carbon nitride, and TiO_2_. In the later developments, H_2_ase was integrated with semiconductor nanomaterials for CO_2_ reduction. However, this system has several potential drawbacks such as the porosity of the electrodes being higher than the size of enzymes, instability, sacrificial characteristics, and the requirements of a high precision and advanced technique for isolating pure enzymes. These flaws may restrict scaling up of solar driven CO_2_ reduction. On the other hand, the whole-cell integration (EABs) with photo biocathode is realistically achievable under field conditions with many possible outcomes. The mechanism for generating electrons and protons at anode and their final utilization at cathode are worth to discuss for understanding some critical advantages and disadvantages of solar driven CO_2_ reduction.

### Reactions at photoanode

**Figure 2:**
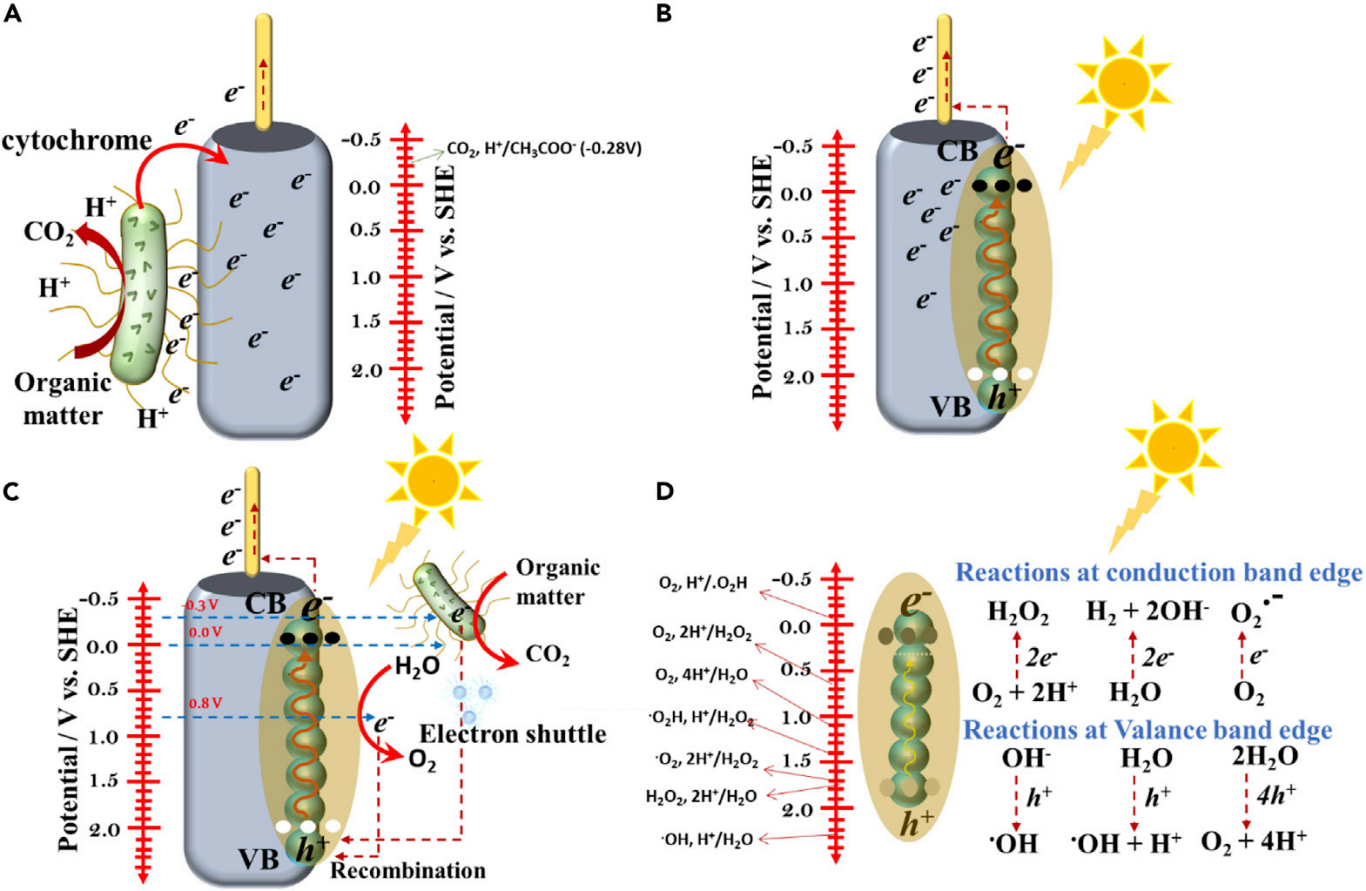
Schematic diagram of different anodes, describing the various mechanisms of major reactions occurring at the interface (A–D) (A) a conventional biotic anode accepting electrons from the EABs and corresponding electrode potential, (B) a conventional photoanode with semiconductor catalyst, (C) a biotic photoanode hybridized between semiconductor material and EABs, and (D) generation of major ROS supported by VB holes and CB electrons at different electrode potentials vs. SHE.

In biosynthesis, availability of H_2_ at biocathode is important. H_2_ serves as a charge carrier for several microorganisms such as proteobacteria, planctomycetes, spirochetes, and euryarchaeota, all capable of fixing CO_2_ (Wang and Ren, 2013; Rabaey and Rozendal, 2010; Rojas et al., 2018). Moreover, H_2_ becomes relatively more important when mixed culture bacterial consortium is used as the source of inoculum. In such a scenario, H^+^ formation at anode via water splitting has been used in many recent studies (Li et al., 2020; Jiang et al., 2020; Noori et al., 2020a). However, the energy demand for water splitting reaction at plain graphite anode is high. This pressing matter is cleverly solved via three distinct methods (Figure 2): (1) a bioanode, which utilizes bacterial metabolism to generate protons (Figure 2A) (Singh et al., 2020), (2) application of a photosensitized abiotic anode, which utilizes solar radiation as an energy source for water splitting (Figure 2B) (Liu et al., 2016), and (3) combining (1) and (2), i.e. a biotic photoanode (Figure 2C) (Fischer and Reviews, 2018). The first method is commonly used in BESs in which EABs oxidize organic matter (preferably acetate) in wastewater, generating electrons and protons. The electrons are transferred to the anode via the cell-wall bounded protein structures such as c-type cytochromes, conductive pili, or via indirect method using redox mediators or both (Torella et al., 2015b; Nichols et al., 2015a; Liu et al., 2016, 2018). Cytochromes are the protein structures consisting of heme factor that acts as the electron transfer agents in the WLP (Figure 1A). Once the electrons are received by the anode, they are transferred to the cathode via an external circuit using either a natural voltage gradient developed between the anode and cathode, or an imposed potential. In both cases, electrons are used as a reducing equivalent to complete the overall redox reaction.

It can be seen from Figure 2A that the bioanode using acetate as the main carbon source derives electrons at an electrode potential of −0.28 V, and these electrons can contribute energy to power the MES system (Lim et al., 2017). At the cathode, electrons and protons are used as the reducing equivalents by EABs to synthesize biochemicals from CO_2_. However, weak bioanodes with high open circuit potential and low current supply are considered to be insufficient to drive cathodic reaction under a fixed cathode potential (Wang et al., 2010). This follows that the same amount of energy needs to be supplied by the anode to cathode to sustain bioelectrosynthesis. Therefore, to maintain the energy demand, abiotic semiconductor-based photoanodes were developed. As the photoanode receives solar radiation equal to or greater than the band gap energy of the semiconducting materials, electrons jump to the conduction band, thereby creating holes in the valence band (Figure 2B). The electrons at conduction band are quickly transferred to the (bio)cathode to compensate for the electrons required for the reduction of CO_2_ to valuable biochemicals. However, the latter method is more interesting as it combines EABs with the semiconductor nanomaterial-treated anodes. On the other hand, EABs deposited with a biofilm at anode generate electrons and protons at lower potential (usually less than the potential of VB of the semiconductor material) by utilizing the organic matter substrate. The electrons then recombine with the holes (at high potential) to regenerate the anode, whereas the protons are transferred to the cathode via the PEM or CEM. The above theory can be fairly understood by the following illustration in which α-Fe_2_O_3_-coated electrode has been showcased as a model biotic photoanode (Figure 2C). The α-Fe_2_O_3_-coated biohybrid photoanodes are popularly used in bioelectrochemical systems — attributed to the favorable band gap (2.1 eV) for light absorption, biocompatibility, high stability at oxidative environment, abundance, and low cost—to derive valuable commodities from the wastewater streams(Fu et al., 2014). At the neutral pH, the band-edge position of α-Fe_2_O_3_ ranges between 0.1 (the edge of the conduction band, CB) and 2.2 V (the edge of the valence band, VB). Hence, holes generated in VB have sufficient energy to receive electrons either from cytochromes of *Geobacteraceae* (Feng et al., 2016) and *Shewanella* (Qian et al., 2014) for examples, or water (discussed later).

The system is certainly beneficial as it can generate electrons and protons at a low overpotential while treating wastewater. Under wet conditions, series of redox reactions occur during photocatalysis (at VB and CB), as depicted in Figure 2D, including some ROSs such as ⋅OH, H_2_O_2_, ⋅O_2_−, and ^1^O_2_ post water-oxidation, and ⋅O_2_−, H_2_O_2_, and ⋅OH post O_2_-reduction (Nosaka and Nosaka, 2017). Considering that the biofilm remains in contact with the sandwiched photoanode (semiconductor-anode current collector base) in PMES, there is a potential risk of damaging the biofilm because of the lethal attack of the ROS species. Most of the ROS species are highly reactive with very less lifetime (e.g. 3.5 μs for ^1^O_2_ in aqueous solution) (Egorov et al., 1989), which can rapidly attack the nearby biomolecules up to a distance of 10 – 400 nm, and also penetrate the cell-membrane of bacteria (Sies and Menck, 1992; Baier et al., 2005). Therefore, it is necessary to control the ROS generation, and also, continuously remove them from the system. Further description of the strategies for the ROS removal is available in section 5.2.

### Reactions at photocathode

**Figure 3:**
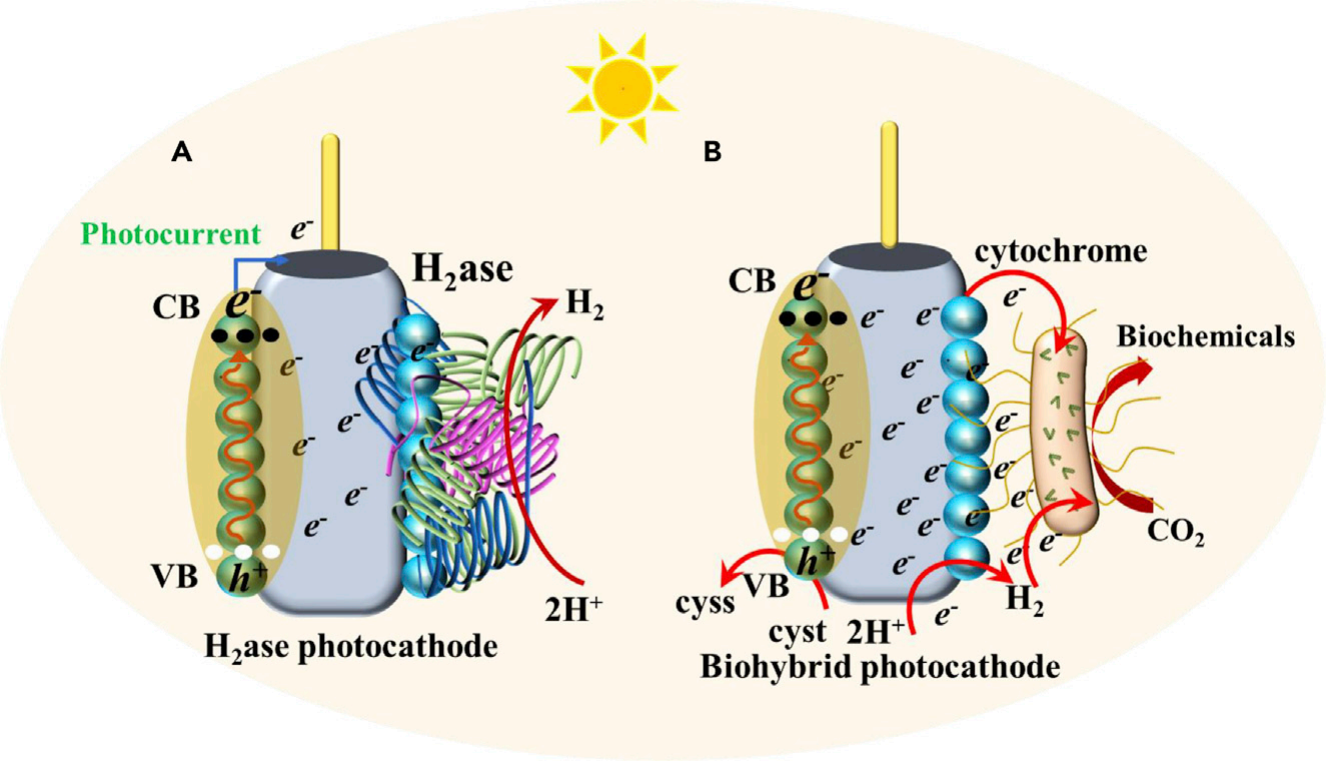
Reactions occurring at different type of cathode in MESs. Charge transfer mechanism in (A) H_2_ase-based photocathode, (B) biohybrid photocathode.

Considering that H_2_ is an electron carrier for EABs, it is important to fix its concentration in the cathodic chamber. H_2_ can be readily made available at cathode by converting incoming protons (from anode) or by reducing water (i.e. H_2_O(*l*) + 2e^−^ → H_2_(*g*) + 2OH^−^) (Cestellos-Blanco et al., 2020). However, in both cases, the overpotential loss for HER is high, and thus an efficient electrochemical catalyst is required to sustain the reaction. Conventionally, Pt-based electrodes have been used to catalyze HER, which may not be deployable because of high fabrication costs (Nichols et al., 2015a). Alternatively, the Ni-foam/carbon nanotube cathode coupled with *S. ovata* was tested in MES for producing acetic acid (Bian et al., 2018). The results demonstrated the substantial reduction in HER overpotential and a high production rate of acetic acid. Similarly, inclusion of graphene in Cu-foam enhanced the conductivity of cathode and promoted HER, which resulted in a high CO_2_ sequestration rate in MES (Aryal et al., 2019). Lately, Kracke et al. developed a robust nickel-molybdenum alloy (NiMo) as the cathode material for MES, which outperformed Pt-based catalyst in producing H_2_ (Kracke et al., 2019). In the recent development, the semiconductor nanomaterial-based biocathodes are interfaced with either pure H_2_ase enzyme or EABs to enhance the H_2_-generation at low overpotential. In the case of whole-cell MES system, single or combination of different pathways, e.g., cell wall bounded H_2_ase, charge transfer proteins (cytochromes), soluble redox mediators, etc. is responsible for H_2_ generation at cathode (Kornienko et al., 2016) (Figure 3A). A photo biocathode provides in-situ electron generation and efficient transfer to the cell membrane, which circumvent additional energy demand to pull the electrons from spatially distant electrode (i.e., anode). Specifically, the electrons and holes are generated in a photocathode under solar irradiation conditions. The electrons are transferred to EAB via the different mechanisms, e.g., direct transfer (cytochromes, conductive filaments), or mediated (H_2_, formate) to ultimately participate in the WLP (Sakimoto et al., 2016; Kornienko et al., 2016) (Figure 3B). The holes recombine with the electrons generated by oxidation of cysteine to cystine, leading to regeneration of the photo biocathode. As discussed above, partial generation of ROS at photo biocathode as well is highly detrimental for the EABs, which can reduce the lifespan of the PMES (Zhao et al., 2018). The details are provided in section 5.2.

The hybrid approach based on combining the semiconducting nanomaterial-based electrodes with biological activity to harvest solar energy is an efficient means for CO_2_ reduction and for generation of valuable chemicals. Currently, the application of PMES has resulted in formation of many useful chemical products from direct reduction of CO_2_ at laboratory scale. However, the PMES systems suffer from the low solar to fuel conversion efficiency, and the reduced selectivity of products. Therefore, understanding the mechanism of semiconducting nanomaterials, reaction kinetics, and interaction between EABs and electrode is important to improve the conversion efficiency and product selectivity of the hybrid systems. The removal of undesirable ROS from the PMES systems and mitigating the toxicity of EABs towards electrodes are the other major challenges associated with the PMES systems (Cestellos-Blanco et al., 2020). The major research focus in this area is on the development of the state-of-the-art solar nanomaterials having the enhanced capture of solar radiation and the increased efficiency of binding with microorganisms for an efficient extracellular electron transport. The surface properties of the materials are mainly tuned to provide a benign environment for the EABs to develop biofilm on the electrodes. In the next sections, we discuss the major recent developments in the semiconductor nanomaterials used as a bioanode, a biocathode or both in PMES.

## Recent development in semiconductor nanomaterials for cathode and anode

**Figure 4:**
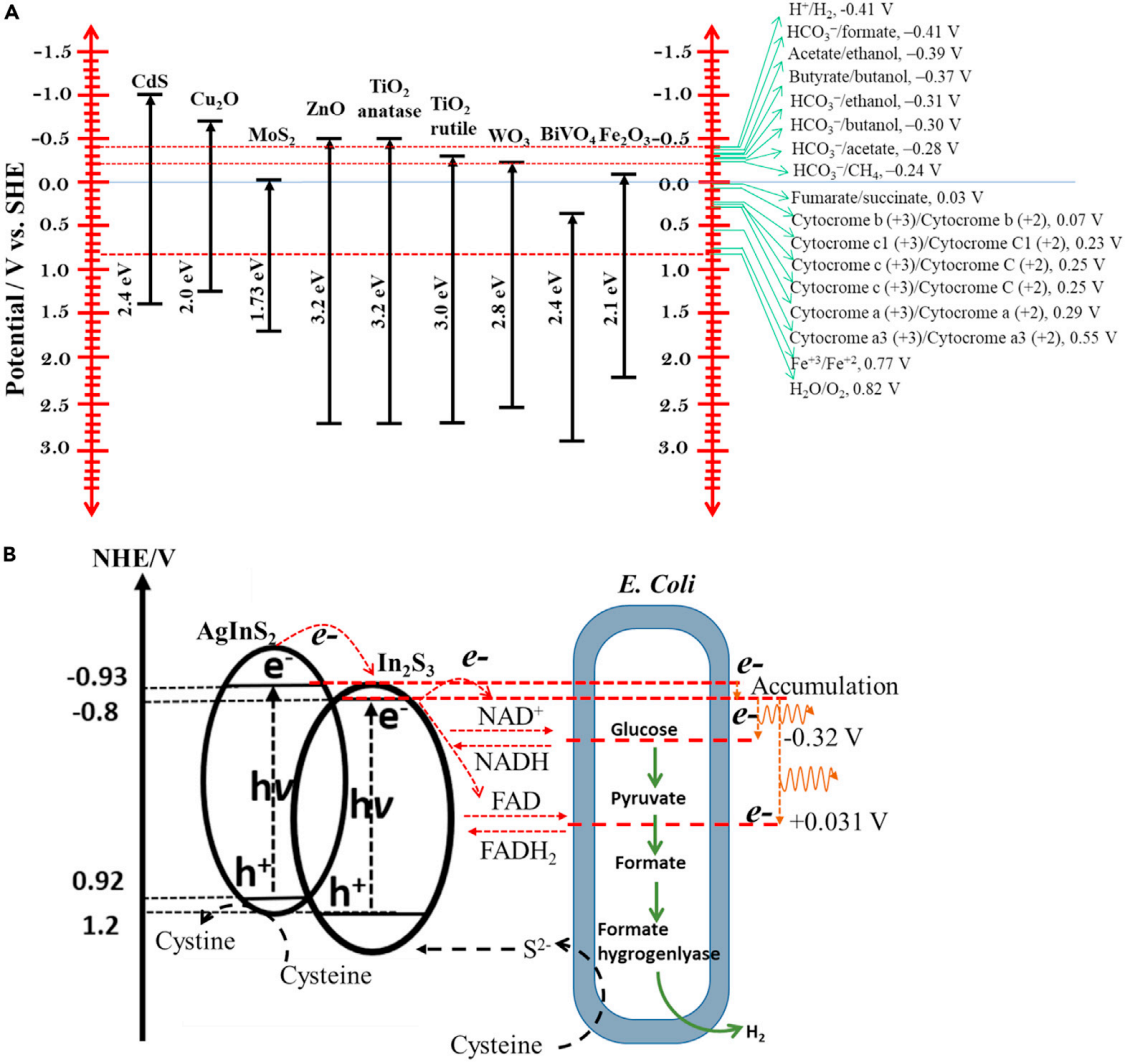
Band alignments in different semiconductor materials and possible outcomes. (A and B) (A) Band edge energy positions of AgInS_2_ and In_2_S_3_ and redox potential of major reactions in PMES system (Part of this figure is reproduced from Tahir and Amin (Tahir and Amin, 2013) with permission of Elsevier), and (B) Electron transfer pathway in AgInS_2_/In_2_S_3_ – *E. Coli* biohybrid system generating H_2_ (reproduced from Jiang et al.(Jiang et al., 2018) with the permission of Elsevier).

As discussed in the earlier section, the EAB-based PMES systems, working on the principles of the conventional MESs, are recent advancements (Claassens et al., 2016). The PMES using the semiconductor-EAB biohybrid anode/cathode is, however, complex because of the involvement of the multiple metabolic pathways. Yet the system is advantageous over the pure enzyme-based (e.g., H_2_ase enzyme) systems. The physicochemical configuration of a semiconductor nonmaterial is of great importance and should possess certain basic criterions as discussed earlier. Many researchers in the past have tried to develop various materials to improve the product yield with an excellent selectivity for biochemical. Especially, the hybrid semiconductor materials (HSMs) — intercalating more than one element—manifest unique properties which can supersede the functionalities of individual components in the hybrid system. HSMs can be synthesized via several precise techniques to generate desirable shape, size, and orientation of each metal in the matrix, with varied properties and applications. The photocatalytic functions of HSMs are extensively investigated in photocatalysis, as systematically presented in the review articles published by Li and Zhang (Li and Zhang, 2009) and Banin et al. (Banin et al., 2014). Nonetheless, there is some scope to discuss further a few special properties of HSMs concerning their applications in PMES systems. A proper arrangement of two materials, e.g., semiconductor and metal, as a single nanoparticle (NP) material allows customizing the band alignment to match the redox potential of the desired redox reactions. There is a perfect thermodynamic relation between the band edge energy positions of a semiconductor material and the redox potentials of the associated chemical species. The band edge position of the photocatalysts must straddle the redox potential of the redox couples to derive redox reactions—a few examples of redox reactions and band edge positions of some conventional semiconductor materials are schematically described in Figure 4. As an example, to derive O_2_/H_2_O and H^+^/H_2_ redox couples, the VB and CV band edge energy positions of the photocatalyst straddle 1.23 V and −0.42 V, respectively (Zhang and Dong, 2019).

In the case of PMES the position of band edges becomes important and should be tuned to straddle with the redox potentials of cytochromes and the metabolic mediators to promote efficient electron transfer. It can be clearly seen from Figure 4A that the reduction of holes generated during photoexcitation of semiconductor materials (most frequently, the material that is used) is thermodynamically feasible via several electron transfer pathways (cytochromes, electron shuttles) in EABs. This can make the charge separation process efficient in biotic photoelectrodes. However, in the case of some semiconductor materials, the conduction band edge energy position falls short (towards more positive) to derive some specific reduction reaction. Thus, the band edge energy gap should be tuned in order to comply with the thermodynamic reduction potential of desired reduction in an unbiased PMES system (without having an external power source). One of the major advantages of HSMs is their abilities to promote rapid charge transfer during photo excitation that eventually leads to the efficient charge separation. In HSMs, the Fermi level of metal is located within the band gap of the semiconductor materials. Fermi level is a thermodynamic quantity (*µ* or *E*_F_) which denotes the work done by a solid body to introduce one unit of electron to the body. It is related to the band energy of a semiconductor material, and the difference between Fermi levels can be measured using a voltmeter (see Figure 1B). The photo exited electrons at the CB of the semiconductor are quickly transferred to the energy levels of metal (Rawalekar and Mokari, 2013). Therefore, metals in the hybrid system act as an electron-sink. The charge separation time is significantly improved in the presence of a hole-scavenger. For example, in an abiotic system Berr et al. used a CdS-Pt hybrid photoelectrode and a sacrificial hole-scavenger (Na_2_SO_3_, 0.1 M) (Berr et al., 2012). The authors noted that the system with Na_2_SO_3_ had a charge-separation delay of 8000 ps as compared to 400 ps in the system without Na_2_SO_3_. In a recent experiment, Jie et al. showed a 213% increase of charge-separation delay in the *M. barkeri*-Ni(2.00%):CdS biohybrid system, as compared to the control HSM electrode (i.e. Ni(2.00%):CdS) (Ye et al., 2020). Interestingly, the hole scavenger used in the second example is sustainable in contrast with the sacrificial (Na_2_SO_3_) hole scavenger used in the first example. These two examples clearly demonstrate that a biotic photocatalytic system with whole cell bacteria has a clear advantage over the other abiotic and biotic systems with sacrificial hole scavengers such as sulfite (SO_3_^2−^), thiosulphate (S_2_O_3_^2−^), hyphophosphite (H_2_PO_2_^-^), and trymethylamine, etc (Ghosh, 2018).

Artificial band alignment in PMES has also been achieved by rendering heterojunctions to be the light harvesters — i.e., semiconductor—semiconductor nanostructures. Heterojunctions are the interface that develops between two layers of dissimilar semiconductors when they come in contact. For examples, AglnS_2_/In_2_S_3_ enabled efficient electron transfer from the CB of AglnS_2_ (positioned at −0.93 V) to that of In_2_S_3_ (positioned at −0.8 V) and to *Escherichia coli* for efficient H_2_-production (Jiang et al., 2018). In this hybrid system, the photo exited electrons at the relatively lower conduction band edge position of the first semiconductor (AglnS_2_) material quickly accumulated in the CB of In_2_S_3_, which were then transferred to the redox mediators of the *E. Coli* NAD^+^/NADH couple (−0.32 V) and FAD/FADH_2_ couple (+0.034), donating a net positive energy to the bacterial cells (Figure 4B). The hybrid system created a high intracellular reduction potential that stressed on *E. Coli*, and thus dispensed with the reducing equivalents to maintain the redox balance, resulting in high charge-transfer kinetics. At the same time, the holes created in the hybrid system were scavenged by cysteine/cystine redox couples. Also, the low band energy gap is an important feature of HSMs from the point of view of practical applications for PMES under visible light. In the past decade, several HSMs were synthesized which had enlarged band edge positions, and thus they could work in visible light, for examples, 400–650 nm for N-Ce co-doped TiO_2_ (Wang et al., 2011a), 400–600 nm for N-F co-doped TiO_2_ (Huang et al., 2006), and 420–610 nm for the PbS quantum dots deposited over Cu/TiO_2_(Wang et al., 2011b). Recently, several types HSMs have been specially designed for the PMES systems to enhance their efficiencies, which are systematically discussed below, and the comparative performance results are outlined in Table 1.

**Table 1:** Performance of various PMES cells using different anodes, cathodes, and micro-organisms reported in the literature.

Anode	Photocathode	EABs	Products	Product generation (mM/day)	CE (%)	Reference
Carbon fiber brush	p-type Co_3_O_4_ nanorods	Activated sludge	HCOOH	5.73	10.5	(Wu et al., 2020)
Carbon rod	WO_3_/MoO_3_/g-C_3_N_4_	*S. marcescens Q1*	Acetate	3.12	73 ± 4	(Cai et al., 2020)
Pt	n^+^/p-Si/NiMo	*M. barkeri*	CH_4_	13.45	82	(Nichols et al., 2015a)
**Photoanode**	**Cathode**					
TiO_2_ nano wires on FTO	carbon cloth	*Methanobacterium formicicum and Coriobacteriaceae*	CH_4_	1.10	96	(Fu et al., 2018)
TiO_2_/CdS	carbon cloth with chitosan	*Methanobacterium and Hydrogenophilaceae*	CH_4_	53.54	92.7	(Xiao et al., 2020)
TiO_2_/CdS/Cu_2_ZnSnS_4_	carbon cloth with chitosan	*Methanobacterium and Hydrogenophilaceae*	CH_4_	53.06	#	(Xiao et al., 2020)
FTO/BiVO_4_/Mo	ceramic hollow tube/Ni Foam	*Mixed inoculum*	Acetate	0.91	62	(Bian et al., 2020b)
IO-TiO_2_|RuP/BiVO_4_-CoO_x_	IO-ITO|*G. sulfurreducens*	*G. sulfurreducens*	Succinate	0.237	#	(Fang et al., 2020a)
CoPi	NiMoZn	*R. eutropha H16*	Isopropanol	3.59 mM	#	
CoPi	Co-P alloy	*R. eutropha*	fused alcohols	Isopropanol ~10 mM and C4 + C5 alcohol ~1.64 mM	#	
**Photoanode**	**Photocathode**					
TiO_2_ nanowires on FTO	p-InP cathode	*M. barkeri*	CH_4_	0.013	74	(Nichols et al., 2015b)
TiO_2_ nanowires	Si nanowires	*S. ovata*	Acetate	21.02	90	(Liu et al., 2015)

CE - Coulombic efficiency; CoPi - cobalt phosphate; FTO - fluorine-doped tin oxide; IO-ITO - inverse opal-indium tin oxide; Pt - platinum

### Photoanode materials

Application of a photoanode for deriving the reducing equivalences (e.g., electron and protons) using water-splitting reaction in PMES is could be a sustainable and practical approach to derive CO_2_ reduction reaction at cathode. However, the photoanode semiconductor materials should be able to capture visible light spectrum to avoid unnecessary UV exposure and efficiently separate the electrons and holes. TiO_2_ NP-based photoanodes have been a prime choice for several years because of their high photochemical stability, low cost, non-toxicity, and reliability (Wang et al., 2014; Luo et al., 2017; Zhao et al., 2015). However, disordered crystal structure of TiO_2_ substantially increases electron transport resistance within the crystal networking because of the random arrangement of crystallites, which impedes the performance of the photo-assisted bioconversion processes (Bierman et al., 2009). Therefore, one-dimensional TiO_2_ nanostructures such as nanotubes, nanowires, and nanorods have been precisely investigated as photoanodes in different devices including PMES, owing to their high electron transport and light scattering abilities (Liao et al., 2012). Majorly, for applications as photoanodes in the PMES systems, rutile TiO_2_ nanowires arrays were synthesized in the solution-phase on fluorine-doped tin oxide (FTO) glass as photon collector and electron conductor because of the low degree of lattice mismatch (< 2%) between the FTO substrate and rutile TiO_2_ lattice structure that allows a high degree of electron transfer kinetics (Ghosh, 2018). For example, Liu et al. (Liu et al., 2015) used the TiO_2_ nanowire-coated silicon (Si), and Nicolas et al. (Nichols et al., 2015a) applied an *n*-Ti O _2_ nanowire/FTO as photoanode to generate electrons and protons via water-splitting reactions. Similarly, a TiO_2_ nanowire array coated on FTO photoanode demonstrated high photocurrent density (Fu et al., 2018). Interestingly, upon coupling this photoanode with the mixed bacterial consortium-enriched biocathode, the PMES system could produce CH_4_ from CO_2_ at a remarkable efficiency of 96%. However, TiO_2_ shows two major drawbacks, which may limit the practical applications of the TiO_2_ nanostructure based photoanodes. First, the electron and holes coexist in the TiO_2_ particles, and hence their recombination probability is high i.e., less charge separation time. Second, and possibly, the most important, TiO_2_ can capture radiation near the UV-light wavelength (< 400 nm), and UV spectrum is potentially harmful for bacteria and human operators. On the other hand, only 5% solar terrestrial radiation is composed of UV radiation (Jiang et al., 2018), which implies that a PMES with TiO_2_ cannot be efficiently operated under direct solar radiation. In this context, the low-band gap semiconductors coated over TiO_2_ nanomaterials can be used over a wide spectrum of solar radiation (as discussed in section 3).

To address this challenge, Xiao et al. (Xiao et al., 2020) coated a thin layer of CdS with a band gap of 2.4 eV on the TiO_2_ nanowire array/FTO substrate. The CdS-TiO_2_/FTO hybrid photoanode demonstrated a perfect band alignment, which extended the light adsorption ability of the photoanode towards visible light (550 nm wavelength much higher than that (< 400 nm) of TiO_2_). The photocurrent-onset potential of the CdS-TiO_2_/FTO photoanode was measured to be around −0.95 V (vs. SHE) as compared to −0.51 V (vs. SHE) for TiO_2_/FTO. The negative shift in the onset potential for photocurrent generation signifies the negative shift in fermi level and a high charge separation because of the inclusion of CdS, which has a lower band gap as compared to TiO_2_. Precisely, the negative shift in Fermi level towards conduction band in a semiconductor material occurs because of the presence of the more negatively than the positively charged particles. Therefore, the CdS-TiO_2_/FTO photoanode is potentially viable to deliver high photocurrents to the biocathode for CO_2_ reduction to CH_4_ (Table 1). In the same study, an improved material, Cu_2_ZnSnS_4_, (band gap 1.5 eV) was also included in CdS-TiO_2_/FTO hybrid system to enhance the light capturing efficiency under visible light spectrum (> 550 nm). Interestingly, this visible light responsive hybrid photocatalyst demonstrated a solar to fuel conversion efficiency of approximately 1.28%, which is six times higher than that of the natural photosynthesis system (Berberoğlu and Pilon, 2010). Moreover, this efficiency was also noted to be significantly higher than that of the traditional photobioreactors producing biohydrogen using monocultures of *Chlamydomonas reinhardtii* algae (0.061%) and *Rhodobacter sphaeroides* bacteria (0.054%) (Berberoğlu and Pilon, 2010). However, it will be early to conclude that the PMES system is better than traditional photobioreactors in terms of the solar to fuel production efficiency. More such studies in this line will be required for a rational comparison, and finding a better device for driving useful chemicals using solar energy in the future.

In the line of making hybrid photoanode materials, recently, Fang et al. (Fang et al., 2020a) developed the highly porous light harvesting opal-indium titanium oxide (IO-TiO_2_) solid electrode via cross-assembly of polystyrene beads as the template, and the TiO_2_ NPs (∼50 nm) as a light-capture catalyst. The electrode was treated with phosphonated [Ru^II^(2,2'-bipyridine)_3_] (RuP)-based dye and used as an abiotic photoanode in a PMES. Under solar radiation, the RuP dye molecule VB electrons electrons and donated to the conduction band of the TiO_2_, which were finally transferred to the IO-TiO_2_—*G. sulfurreducens* biohybrid cathode through the external circuitry to produce succinate from fumarate. The reduced form of RuP is then regenerated at the photoanode via counter oxidation by triethanolamine (TEAO). However, TEAO is a sacrificial chemical, and thus, the assembly is not suitable for practical systems. To overcome this drawback, the authors, in the same study, developed a BiVO_4_-CoO_x_ nanostructure based photoanode that omitted the need for sacrificial chemicals to scavenge the holes. BiVO_4_-CoO_x_ is an excellent photocatalyst with remarkable properties owing to its heterojunction structure developed via the interaction of the n-type BiVO_4_ and the p-type CuO_x_ (Kanigaridou et al., 2017). This special property of BiVO_4_-CoO_x_ allows efficient charge separation at p-n heterojunction upon receiving the light photons.

The cobalt-phosphate (CoPi) photoanode-based water-splitting system has also demonstrated promising results with low photodamage during the operational cycles (Kanan et al., 2009). Therefore, CoPi is considered to be the self-healing photocatalyst and has been used as a reliable photoanode in PMES (Liu et al., 2016; Torella et al., 2015b). However, in an abiotic photoanode, efficient charge separation is always a challenge. As discussed earlier (see section 2.1), the electrons generated by EABs at photoanode (biohybrid photoanode) recombines with the holes, leaving the electrons at CB free to transfer to the biocathode. Therefore, the photocurrent density can be increased further. In an earlier study, the α-Fe_2_O_3_-*Shewanella* biohybrid photoanode demonstrated a surprisingly 150-times higher photocurrent as compared to the α-Fe_2_O_3_ photoanode (dead cell) (Qian et al., 2014). Similar observation was also noted when Feng et al. (Feng et al., 2016) hybridized α-Fe_2_O_3_ with mix anaerobic bacteria and used it as a photoanode. They noted a significantly higher photocurrent as compared to the α-Fe_2_O_3_ photoanode without bacteria due to excellent scavenging of holes.

### Photocathode materials

A photo biocathode operates on the less-energy consuming pathway, as it can provide the reducing equivalence (electron) onsite to EAB for the targeted synthesis of biochemicals from CO_2_. However, this concept is rather new and there are challenges that need to be addressed. For example, the photocathode should have an adequate biocompatibility, high CO_2_ adsorption capacity, and should show the low or zero production of ROS. The one-dimensional semiconductor nanostructures, for examples, nanorod, nanowires, and nanotubes showed relatively less CO_2_ mass transfer losses, with improved biocompatibility (Liao et al., 2011; Imani et al., 2016). In the first demonstration of the EAB-based PMES, the silicon-TiO_2_ nanowires composite cathode was interfaced with *S. Ovata* to synthesize acetate from CO_2_ (Liu et al., 2015). Interestingly, when they incorporated Pt on the silicon-TiO_2_ nanowires (HSM), the PMES could be operated under aerobic conditions (21% O_2_, 10% CO_2_, 69% N_2_ in the head space). Such operation was possible because of the efficient scavenging of O_2_ by Pt by promoting oxygen reduction reaction (ORR) at the interface. By using this type of catalyst, the O_2_ poisoning in the biocathode can be reduced. Moreover, it will be possible to apply this cathode in a single chamber PMES system, which is a convenient design for field scale studies because of the less fabrication cost and ease of maintenance (Gavilanes et al., 2019). In a later development, the TiO_2_-passivated n+/p-Si was covered with a thin layer of Ni-Mo alloy using the sputtering coating, and was used as the photo biocathode in a PMES coupled with the Pt-coated abiotic anode (Nichols et al., 2015a). The Ni–Mo/TiO_2_- n+/p-Si hybridized *Methanosarcina barkeri* was able to capture visible light (740-nm) and demonstrated a low overpotential reduction of CO_2_ to CH_4_ as compared to earlier designs. Interestingly, this hybrid cathode does not impart any toxicity on the metabolism of *M. barkeri.* Recently, Wu et al. (Wu et al., 2020) fabricated a photo biohybrid cathode with the p-type cobalt (II,III) oxide (Co_3_O_4_)/mesoporous nickel (Ni) foam as the solar collector and EAB as the biocatalyst for an efficient conversion of CO_2_ to formic acid (HCOOH) in PMES (Table 1). As discussed earlier, the semiconductor-metal based hybrid photoelectrodes are better than the single semiconductors in several ways. In this study also, the Co_3_O_4_/Ni hybrid demonstrated excellent band alignment and favorable band edge positions (VB at 2.62 eV and CB at 0.23 eV) to derive HCOOH from CO_2_. In addition, the hybrid catalyst had a uniform particle size of 80 nm size with the specific surface area of 40.7 m^2^/g, which allowed effective adsorption of CO_2_ molecules at the reaction interface, lowering the mass transfer losses. The authors also integrated a carbon fiber brush bioanode in PMES as the secondary source of reducing equivalence to operate under dark conditions. Such design can be particularly useful in two distinct ways: 1. It can operate in dark conditions with no or minimum light source and 2. The additional electrons generated at bioanode by endogenous activities of EABs can act as additional hole scavenger during daytime. After absorbing light energy, the photo-generated electrons at cathode possess higher potentials as compared to the electrons generated at bioanode. Thus, the electrons from bioanode easily combine with the holes at photocathode (reaction 3). Hence, the electrons generated at bioanode enhance charge separation at photocathode and increase the electrocatalytic ability of Co_3_O_4_ and reduce CO_2_ to HCOOH (reaction 4). Separation of photo-induced electrons from holes is the governing factor for improving the reaction kinetics and CO_2_ conversion efficiency of PMES (Kornienko et al., 2018). The reactions in the bioanode and photocathode chambers of PMES are described as follows.

(Equation 1) Bioanode: CH_3_COO^−^ + 2 H_2_O → 2 CO_2_ + 7 H^+^ + 8 e^−^

(Equation 2) Photocathode: Co_3_O_4_ + hv → h_vb_^+^ + e_cb_^–^

(Equation 3) e^−^ + h_vb_^+^ → hv

(Equation 4) CO_2_ + 2 H^+^ + 2 e_cb_^−^ → HCOOH

In another improved anode/cathode coupled PMES hosting *Serratia marcescens Q1* electrotroph, the graphitic carbon nitride (g-C_3_N_4_) decorated with tungsten oxide (WO_3_) and molybdenum trioxide (MoO_3_) NPs were used as a photo biohybrid cathode, and a plain carbon rod was used as a bioanode to produce acetate (Cai et al., 2020). Formation of Z-scheme heterojunction between WO_3_/MoO_3_ and g-C_3_N_4_ enhanced the interfacial contact area and increased absorption of solar light. Besides, the WO_3_/MoO_3_ and g-C_3_N_4_ had some remarkable properties, e.g., thin catalyst layer ∼2.5 mm thickness, high conductivity (103 mS/cm) and a large negative potential (- 1.1 V vs. SHE) that enhanced the charge separation, and electrocatalytic activity for H_2_-generation, which eventually enhanced the rate of acetate production (Table 1). Also, the photo excited electrons combined with H^+^, producing H_2_ that was further used as an electron carrier for *S. marcescens* to reduce CO_2_ to acetate.

From the overview presented above, it is clear that the generation of photocurrents by semiconducting nanomaterials makes the system self-biased to carry out the photoreduction of CO_2_ to valuable chemicals. Till date, only handful of studies are available in this emerging field. More research efforts are needed to address many issues, especially related to photo biohybrid cathode.

## Genetic manipulation of EAB

Study of evolution of EABs, their phenotypic adaptations and genome sequencing can provide an insight into the possibility of genetic manipulations of an microbial entity to carry out the desired organismal functions (Elena and Lenski, 2003). Adaptive evolution is a natural process in which several beneficial mutations are observed in the microorganisms to survive in the specific environment (Rosenzweig et al., 1994). To promote the natural selection and adaptive evolution, the electron-donor and the carbon-source can play a vital role in altering the metabolic pathways and changing the genetic information of an EAB. For example, Fang et al. (Fang et al., 2020b) noted the transcriptional gene expression in *G. sulfurreducens* grown in response to the available electron donors to align their metabolism with physiological requirements. They found down regulation in the genetic modulation of *G. sulfurreducens* grown on the electrode surface as compared to the fumarate-based culture medium to save the energy requirement for metabolism.

Besides the natural section, beneficial genetic mutation can also be artificially induced via gene deletion and combination techniques. For example, an homoacetogen, *Clostridium ljungdahlii* was genetically modified for production of biofuels using MES (Leang et al., 2013). The manipulation of genomic structure through gene deletion in *C. ljungdahlii* explained its ability towards developing the basic building block for formation of biofuels. Deletion of a non-desirable competing gene, called as aldehyde dehydrogenases (*adhE1),* responsible for producing aldehydes during CO_2_ reduction enhanced the production of targeted acetate. However, transforming genetic information of EABs using plasmids (extrachromosomal DNA molecule within a cell) of different bacterial strains is a relatively recent approach and requires further research. The genetically manipulated autotrophic acetogens such as *Acetobacterium woodii* selectively produce acetate by increasing carbon flow in WLP (Straub et al., 2014). In this approach, *A. woodii* was modified with plasmids (pJIR750 THF and pJIR750 pta-ack) derived from a *Clostridium acetobutylicum* ATCC 824, which enhanced the production rates of acetate relative to the reference strain.

Thus, the plasmids incorporated in the genes of *A. woodii* enhanced its capability of CO_2_ fixation by overexpressing enzymes of WLP. Similarly, *E. coli* was modified with different plasmids to produce more selective products such as n-butanol, polyhydroxybutyrate biopolymer and three isoprenoid compounds (Liu et al., 2015). In another study, the wild-type *Ralstonia eutropha* was modified using plasmid pEG12 resulting in the high selectivity and production rates of isopropanol (Torella et al., 2015a). The modified form of *R. eutropha* (*Re*2133-pEG12) introduced four genes responsible for directing the pathway of acetyl-CoA towards the production of isopropanol at an efficiency of ∼90%. Therefore, either the introduction of foreign genes or deletion of genes responsible for the generation of undesirable products seems to be promising for the production of desired biocommodities via CO_2_ reduction.

## Way forward

Bioelectrochemical systems based on the bacterial metabolic pathways as a model reaction to derive renewable energy from waste resources is demonstrated as a potential solution to global energy shortage (Noori et al., 2018, 2019; Das et al., 2018; Rajesh et al., 2018). For example, a microbial fuel cell can generate electricity from organic matters present in waste streams wiring EABs with graphitic or metal electrodes (Noori and Verma, 2019; Reddy et al., 2019; Noori et al., 2016). Reversing the metabolic pathways of EABs can generate even more interesting products such as methane, ethanol, butanol, acetic acid, biopolymers, pharmaceuticals, etc. at biocathode from CO_2_ as sole carbon feedstock (Sadhukhan et al., 2016, May et al., 2016; Roy et al., 2016). In the recent developments, natural photosynthesis machinery is mimicked in laboratory via intervention of the cutting-edge semiconductor biohybrid cathode materials in MESs. However, this improved hybrid system is not fully developed yet, and several shortcomings persist, which need to be addressed to make the technology commercially competitive with the existing solar technologies.

### Semiconductor nanomaterials and electrodes

The semiconductor nanomaterials with low energy band gaps (typically around 1.5 – 2 eV) are required to carry out solar-driven CO_2_ reduction in PMES to compete with the existing solar technologies. Till date, only handful of such electrode materials are developed to comply with the required band-gap regime. To overcome this difficulty, formation of “Z-scheme” using suitable combinations of semiconducting nanomaterials (i.e., hybrid materials) with appropriate bandgaps and band edge energy positions seems to be practical for the PMES applications. Selection of such semiconducting nanomaterials should also fulfill two requirements: flux generated by photoexcited energy carriers from semiconductors should be high enough to meet the current consuming demand of biocatalyst, while providing sufficient photovoltage at the same time for the reactions to proceed efficiently. In addition to the efficient photocatalytic properties, the materials should necessarily have excellent surface properties to host EABs, such as biocompatibility, stability, high surface area, active sites for bacterial adhesion, charge mobility, and electron diffusion rate (Gizzie et al., 2015, Young et al., 2012). For examples, semiconductor nanowires, NPs, and nanorods (Si-based, TiO_2_, WO_3,_ etc.) have shown remarkable optical and electronic properties because of the quantum confinement effects at nano scale, and they also possess high surface area (Chen and Wang, 2014). Quantum confinement can be define as the change in the optical and electronic properties of a material when the material sample size is less than 10 nm. One of the immediate effects of the quantum confinement is the increased band gap as shown in Figure 1E (Corcoran, 1990). Therefore, their applications in PMES system could be beneficial to improve the efficiency. However, the band gap and band edge energy positions should be appropriately leveled to derive desirable reactions. Recently, the organic dyes-inorganic hybrid electrodes (specially, RuP-TiO2 hybrid) have been investigated in several biological systems, which efficiently worked in visible to near infrared region, and showed high charge separation ability (Watanabe, 2017)(Qin and Peng, 2012; Monari et al., 2011; Reisner et al., 2009; Caputo et al., 2015). These materials demonstrated both directional charge transfer (dye to metal and vice versa) either via direct or the redox mediators (e.g., methylviologen dication, MV2+) by simply mixing the components in the reaction solution(Thompson et al., 2013). However, mas transfer limitation in such systems cannot be avoided (Tran et al., 2012; Willkomm et al., 2016). Hybridization of organic dyes with metal components via the covalent bonding or the other surface interactions can avoid such limitations, and improve the charge transfer efficiency by orbital overlapping and stability in the environment (Kruth et al., 2013).

The physiology electrode surface is also very important that provide an active surface to interact with biological moieties. Therefore, understanding of biotic-abiotic interaction (enzyme/microbe-electrode) is key to success of the process. In case of pure enzyme as a model biotic charge sink, it is easy to manipulate the electrodes as the size, surface charge density, and hydrophilicity/hydrophobicity of an enzyme are already known. For example, the larger subunit of H_2_ase enzyme has a minimum radius of ∼4 nm, and therefore, it can be easily adsorbed in the electrodes having pore size of 5 nm – 5 μm (Erickson, 2009; Schilter et al., 2016). However, enzymatic PMES is not a viable option because of several reasons as discussed in section 2. By contrast, the physical footprints of EABs are much larger (∼1 μm^2^) than that of the enzymes that could lead to inaccessibility of the electrode with pore size less than the physical foot prints of EABs (Kornienko et al., 2018). Therefore, it can be a critical bottleneck from the point of view of making a good electroactive biofilm on the cathode electrode in PMES. Therefore, the pore size of electrode must be tuned for its high accessibility to the EABs. In line with the efficient electrode development, genetic advancement in the EAB is also equally important to achieve an economically viable product generation from PMES. The physiology of EABs could be upgraded by genetic engineering to communicate with the electrode via the improved cell-wall-bounded enzymes and self-secreting redox mediators, e.g. flavins (Kormányos et al., 2016; Von Canstein et al., 2008; Marsili et al., 2008) (Zhang et al., 2018). Genetic mutation of EABs using strong mutagenic agents such as nitrosoguanidine and ethyl methanesulfonate, followed by genome shuffling could be useful to derive metal and ROS resistant genes with high electrochemical activity (Pastorella, 2014).

### Reactive oxygen species and CO_2_ solubility

**Figure 5:**
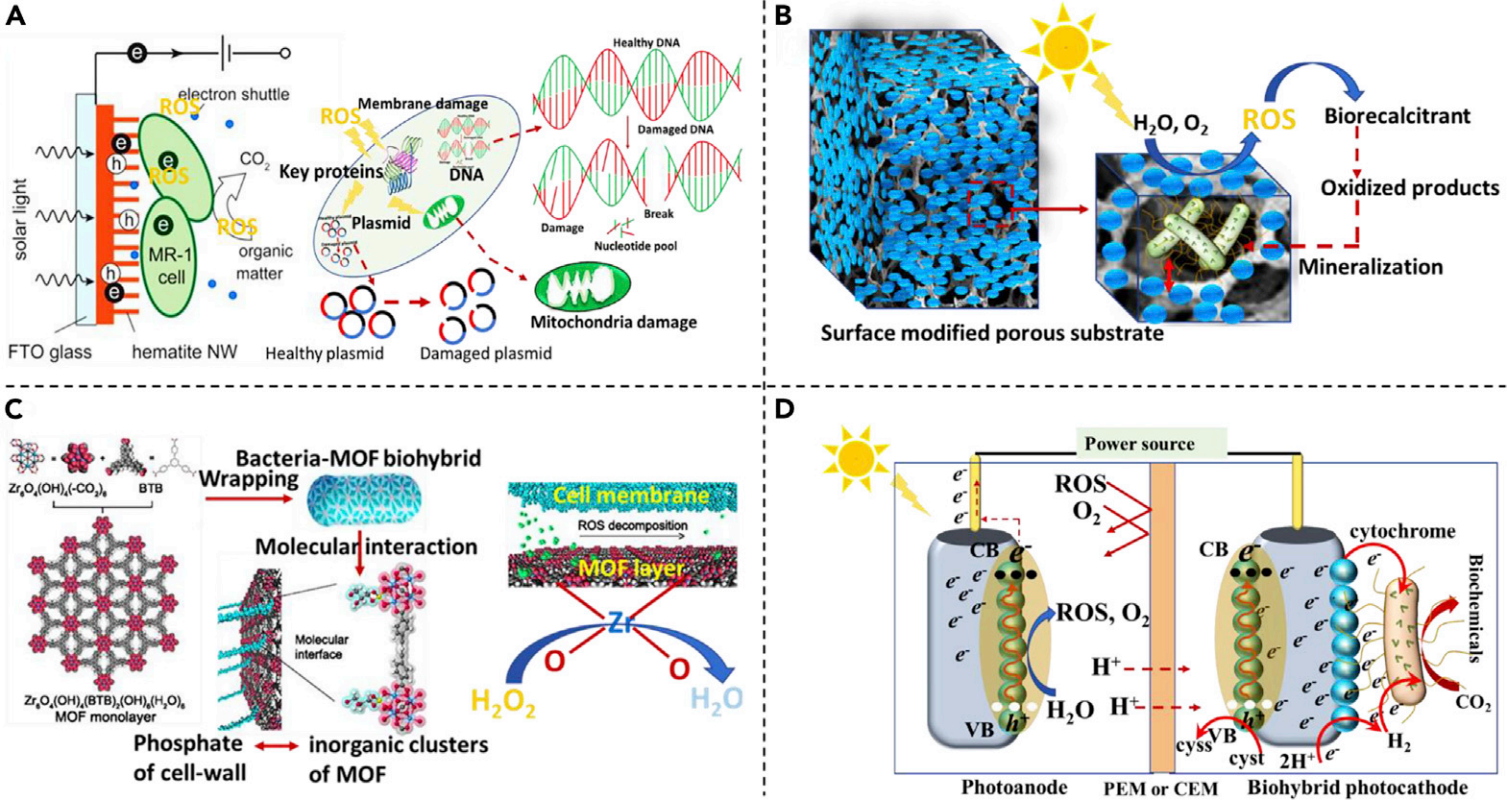
Generation of ROS in PMES and the mitigation strategies. (A–D) (A) Generation of ROS in biotic photo electrodes and their lethal effects on bacteria (a part of this figure is reproduced from Qian et al., 2014 (Qian et al., 2014) with kind permission of American Chemical Society, Copyright © 2014), (B) ROS management in ICBP systems using highly porous substrate (foam-based electrodes), (C) metal organic framework (MOF) used as a cytoprotective layer on anaerobic bacteria adapted from Ji et al. (2018) (Ji et al., 2018b). Note: this figure is slightly modified from original figure, and (D) a dual chamber PMES system inhibiting cross-over of ROS and O_2_ from anode chamber to cathode chamber via a PEM or CEM.

Intermediate generation of cascade of ROS at solar bioanode or biocathode is highly detrimental for the EABs, causing serious damage to the plasma membrane and deactivation of the microbial metabolism (Zhao and Drlica, 2014; Jeong et al., 2006). Figure 5A shows the major lethal effects of ROS on bacteria and some strategies to scavenge ROS from PMES systems (Figures 5B–5D). An excellent option could be to use the in-situ generated ROS species as an strong oxidizing agent to degrade biorecalcitrant aromatics such as trichlorophenol, dinitrotoluene (Yang et al., 2019; Sablas et al., 2020; Gonçalves et al., 2020) using “Intimate coupling of photocatalysis and biodegradation (ICPB)” method (Marsolek et al., 2008). In the ICPB technique, biofilm is cultivated inside the cubic porous solid substrate while outside (exposed to the light) is covered with a thin layer of semiconductor materials (Figure 5B). In this way, ROS generated at surface diffuse through the pores and harm the biofilm located away from the light exposed surface, while at the same time, biorecalcitrant materials can be degraded and mineralized by bacteria. Using this technique, recently, 4-chlorophenol (4-CP) was successfully removed in biotic photoanode (with N-doped TiO_2_ semiconductor) while increasing the photocurrent generation (Zhou et al., 2017). Apart from that, cell membrane engineering is demonstrated as an innovative solution. Recently, Ji et al. (Ji et al., 2018a) wrapped *M.* thermoacetica with the monolayer of (1 – 2 nm) zirconium metal organic framework (Zr-MOF) and applied in a PMES (Figure 5C). They observed five-times less death of the EABs wrapped with Zr-MOF as compared to the EABs without any such wrapping. Initially, the generated ROSs were converted to hydrogen peroxide (H2O2) on the top layer, and then decomposed by Zirconium oxide subunit of MOF, thus resulting in the complete quenching of ROS from the system (Karnholz et al., 2002). Additionally, excess Zr-MOF allowed protection to the fresh grown cells formed after reproduction of EABs, thereby showing the great potential of MOF toward increased lifetime of EABs in the oxygen environment. In line with the Zr-MOFs, more materials need to be developed and tested in a variety of bacterial species. It is worth to mention here that the MOFs are highly tunable with excellent physicochemical properties, emerging as a miracle material for gas separation, energy, and environmental applications (Safaei et al., 2019; Li et al., 2017; Das et al., 2020). High specific surface area and CO2 adsorption capacity makes it unique for future application in PMES (Morozan et al., 2012). It is also suggested that the anode and cathode should be separated using a PEM or CEM that would prevent migration of oxygen or ROS to the cathode chamber (Chang et al., 2016) (Figure 5D).

Adsorption of CO_2_ in a typical electrolyte solution is very low (∼25.11 mM-C), and also the activation of CO_2_ for reduction requires a higher amount of energy as compared to H_2_O (Vassilev et al., 2018). Therefore, in a close system, where CO_2_ reduction reaction is targeted, reduction of H_2_O is the kinetically favorable reaction (Chang et al., 2016). This action ultimately hampers the product selectivity. Also, it leads to the low accessibility of CO_2_ to the microbial active sites, eventually hampering the performance. In some earlier studies, this challenge was addressed by continuously purging an excess amount of CO_2_ in the electrolyte (Bajracharya et al., 2017), circulating CO_2_ in the overhead (Mateos et al., 2019), and adding sufficient quantity of bicarbonate as the CO_2_ source (Zaybak et al., 2013). These techniques seem to be useful to mitigate CO_2_ depletion, but the original challenges persist. The in-situ adsorption of CO_2_ on the gas diffusion electrodes can be a feasible option. For example, Srikanth et al. (Srikanth et al., 2018) fabricated a dual layer VITO-Core gas diffusion electrode having activated carbon and polytetrafluoroethylene (PTFE) layer on either side. They reported enhanced CO_2_ solubility on the activated carbon layer, which in turn sufficiently enhanced product formation. Similar electrodes with an additional layer of semiconductor nanomaterials can be developed to enhance the CO_2_ solubility and reduction rate.

### Other issues

An excess production of acetic acid can sharply decrease the electrolyte pH, leading to decrease in the microbial activity. At low pH, the selectivity of C2, C4, and C6 carboxylic acids and their corresponding alcohols were noted low (Prévoteau et al., 2020). Hence, the pH of the electrolyte needs to be balanced periodically. Besides, the end products such as butyric and hexanoic acid are toxic to some of the microbial species (Candry et al., 2018). These acids are produced from acetate and ethanol after a complex sequence of reactions. Thus, strategies to separate the products should be developed to maintain a high product efficiency and long operational time. Moreover, it is also desirable that the generated photoelectrons are completely utilized by the electron generated by EABs avoid occurrence of the harmful parallel reactions such as water-reduction and/or generation of ROS (Nosaka and Nosaka, 2017). In that case, tuning in the position of valence band edge position of photocatalyst with the redox potential of cytochromes or mediator could be a feasible option.

## Outlook and future prospective

**Figure 6:**
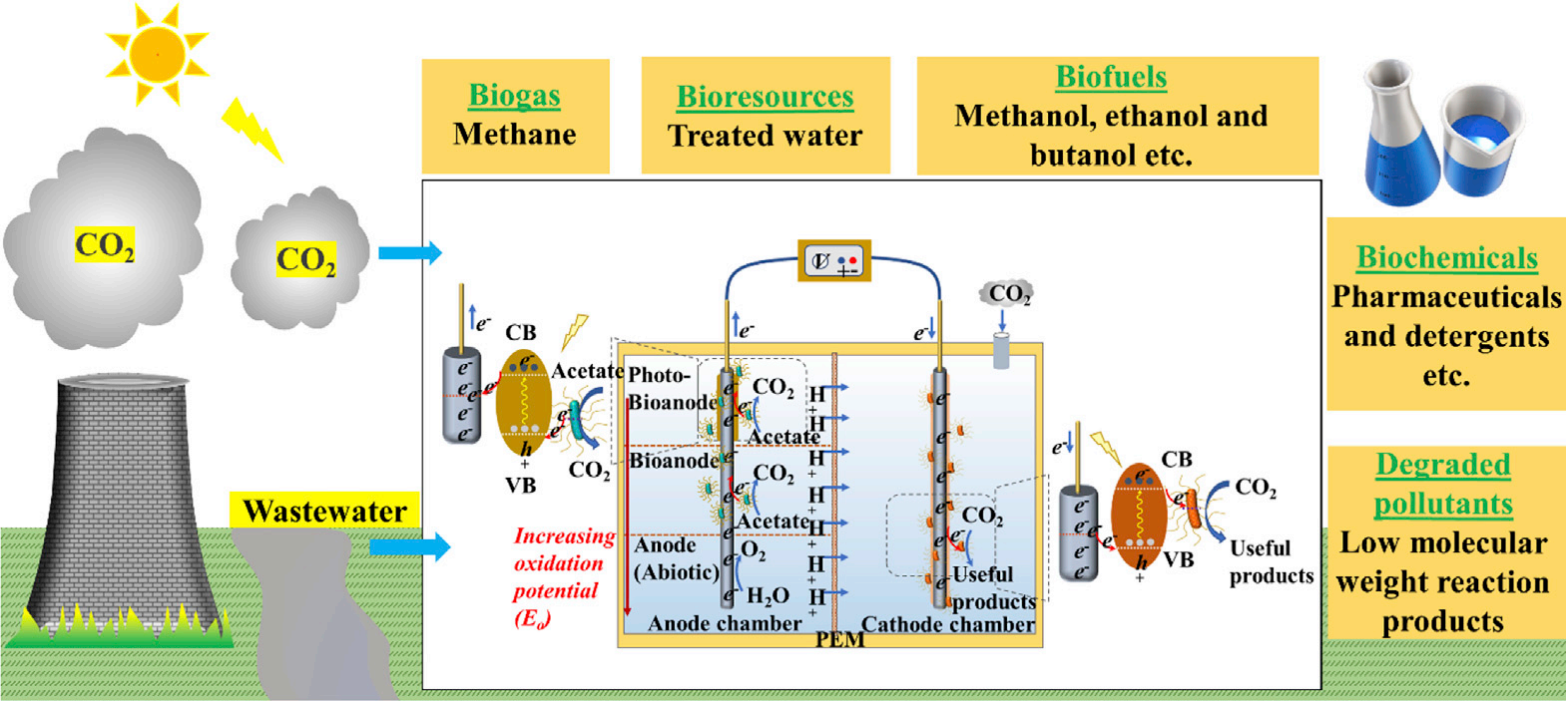
Overall advantages of PMES to attain environmental sustainability and develop a carbon neutral circular economy

Production of valuable chemicals and low carbon fuels using renewable sources of energy via the intervention of MES system can promote environmental sustainability and carbon neutral bioeconomy, as depicted in Figure 6. CO_2_ emission in the environment is highly dangerous for the global environment. Thus, development of CO_2_ capture and conversion technologies are necessary. In this scenario, PMES system could be an attractive solution, which provides us an opportunity to drive green fuels from CO_2_ at a competitive price. Recently, Salimijazi et al., nicely analyzed the engineering constraints of MES and provided the model-based solutions, reaching at least 50% electrical to biofuel conversion efficiency in PMES (Salimijazi et al., 2020). However, this process needs substantial development before its application in real condition. A few major challenges include judicious selection of biocatalysts, wiring of EABs with the electrodes, low solubility of CO_2_, intermediate ROS generation, and an optimization of the reaction parameters such as pH of reaction medium, intensity of light irradiation, flowrate of CO_2_, and tuning of band gap of semiconducting electrodes. The efficient interaction of EABs with electrode is considered to be the most influential parameter, which determines the charge flux and eventually the process efficiency. The properties of electrodes, semiconductor materials, as well as EABs have equal contributions in the interaction process. Hence, the selection of electrodes, semiconductor materials, and the EABs requires a careful review. In this context, electrodes with improved surface properties (viz., high surface area, biocompatibility, and conductivity), HSMs (e.g., metal-semiconductor, semiconductor-semiconductor, organic dye-semiconductor), and the genetically engineered pure strains of EABs could lead the way for future scalable PMESs (Tremblay and Zhang, 2015; Ross et al., 2011). High surface porosity of the electrode will be an additional advantage to adsorb CO_2_ and supply nutrient into the matrix for an easy access to the EABs. In some cases where the maintenance of a complete sterilized system is nearly impossible, it is always better to opt the mixed culture bacterium over pure culture EABs. The mixed culture inoculum contains different types of EAB phenotypes, which are capable of harvesting electrons from the electrodes via all the known pathways, e.g., cytochrome and/or conductive filament, and mediated (H_2_, formate, or flavins). The PMES with mixed culture microbes will be more robust to withstand the environmental and self-generated toxic shocks (e.g., butyric and hexanoic acids and ROSs) (Rosenbaum et al., 2017). Finally, the optimization of various reaction and reactor design parameters can improve the process efficiency significantly.

## Conclusions

In this review, an insight of recently developed semiconductor-based biohybrid materials in connection with photo-assisted MES is presented. Photon energy received from light source excites the valence band electrons to the conduction band in a semiconductor material, which then can be used as a reducing equivalent by the EAB to derive organic compounds from CO_2_. Thus, the performance of PMES systems is dependent on the properties of semiconductor materials. Recently, HSMs have gained considerable attention because of strong interactions of several individual elements, with improved optical (e.g., low band gap and suitable band edge energy positions) and electronic properties (e.g., high charge mobility and charge separation), which also efficiently interacted with EABs for charge transfer. Yet, there are several research lacunas in niche area, such as the development of an efficient light harvester, ROS management, product separation, and low CO_2_ absorption in electrolyte, which are to be addressed in PMES before scaling up. This review paper concisely built a strong narrative from the basics of PMES to the more complex functionality of semiconductor-based materials that will be a milestone for further research in this field.

## Limitations of the study

Authors did not notice any limitation of the study since no experiment was conducted.

## Resource availability

### Lead contact

Further information and requests for resources may be addressed to the lead contact, Mohammad Tabish Noori, PhD, E-mail id: md.noori@uah.es, Tel: +34 622825744.

### Materials availability

The review paper did not synthesize any material and reagent.

### Data and code availability

The article did not generate any software or code. Corcoran, 1990
